# Beyond angiogenesis: the paradoxical emerging pro-tumorigenic role of Thrombospondin-2

**DOI:** 10.3389/fphar.2026.1897133

**Published:** 2026-09-25

**Authors:** Hetal Abhay Shah, Maushmi S. Kumar

**Affiliations:** Somaiya Institute for Research and Consultancy, Somaiya Vidyavihar University, Mumbai, Maharashtra, India

**Keywords:** chemoresistance, immune exclusion, invasion, metabolic reprogramming, metastasis, phenotypic plasticity, Thrombospondin-2, vasculogenic mimicry

## Abstract

The tumor microenvironment is a complex ecosystem where the extracellular matrix serves as a dynamic signaling hub and regulates tumorigenesis. Thrombospondin-2 (THBS2), a matricellular extracellular matrix protein, has traditionally been regarded as an inhibitor of angiogenesis. However, emerging evidence reveals a pleiotropic and paradoxical pro-tumorigenic role of THBS2 in several malignancies. THBS2 has been identified as a multifaceted regulator of critical mechanisms driving cancer progression including angiogenesis induction, vasculogenic mimicry, sustained proliferation, invasion, metastasis, phenotypic plasticity, and immune suppression in aggressive cancers. By modulating diverse signaling pathways, especially the PI3K/AKT/mTOR axis, remodeling the collagenous architecture of the extracellular matrix, and formation of non-endothelial vascular channels, THBS2 supports tumor progression and metastasis. This minireview encompasses current research regarding the emerging molecular mechanisms by which THBS2 regulates pro-tumorigenic functions and discusses its role in resistance to therapy. The diagnostic and prognostic value of THBS2, particularly in aggressive cancers, underscores the need for the development of novel therapeutics specifically targeting this deregulated protein.

## 1 Introduction

Cancer is a paramount global public health threat with approximately 20 million new diagnoses and 9.7 million fatalities as per GLOBOCAN 2022 estimates. Moreover, the cancer incidences are projected to rise by 77% to reach 35 million whereas the mortality rate is expected to double by 2050 (Bray et al., 2024). Factors driving this upward trend are not limited to genetic predisposition, lifestyle and environmental risk but also include enormous range of acquired adaptive mechanisms by tumor cells which drive tumor progression and induce chemoresistance (Damani Shah et al., 2020; Ramos et al., 2021). These hallmark biological capabilities acquired by tumor cells include sustained proliferative signaling, growth suppressor evasion, resistance to apoptosis, replicative immortality, sustained angiogenesis, activating invasion and metastasis, genome instability, tumor-promoting inflammation, deregulating cellular metabolism, evading immune destruction (Hanahan and Weinberg, 2000; 2011). Furthermore, the emerging hallmarks of cancer enabled by non-mutational epigenetic reprogramming and polymorphic microbiomes include unlocking phenotypic plasticity and abundance of senescent cells which collectively drive neoplastic progression and therapeutic resistance (Hanahan, 2022). Besides, tumor progression is profoundly shaped by the complex ecosystem of the tumor microenvironment (TME) which comprises an array of stromal cell types such as immune cells, fibroblasts, and vascular endothelial cells. These cells are embedded within the extracellular matrix (ECM) which is a highly dynamic non-cellular scaffold composed of collagens, proteoglycans, and glycoproteins. Rather than merely acting as a passive structural support, the ECM serves as a critical signaling hub governing cell fate by exerting biochemical and mechanical forces (Yuan et al., 2023). Taken together, this implies that cancer progression is dictated not only by the genetic aberrations within the tumor cell but also governed by the biochemical and biophysical properties of the surrounding TME. Among the key players in this niche is Thrombospondin-2, a matricellular trimeric glycoprotein belonging to the thrombospondin family.

Thrombospondins (TSPs) are a family of five evolutionarily conserved glycoproteins with diverse functions. They integrate signals between the ECM and several cell-surface receptors to regulate for vascular remodeling and synaptic assembly (Adams and Lawler, 2011; Bornstein, 2001). THBS2 belongs to the group A thrombospondins, sharing structural homology with Thrombospondin-1 (THBS1). For decades, THBS2 has been regarded as an endogenous inhibitor of angiogenesis known to bind CD36 receptors on endothelial cells inducing apoptosis and inhibiting migration (Bornstein, 2001; Niu et al., 2023). However, a significant paradigm shift has occurred. In many high-grade solid tumors especially, pancreatic adenocarcinoma, lung adenocarcinoma, breast carcinoma, and colorectal cancer, THBS2 acts as a pro-tumoral factor. Furthermore, THBS2 overexpression correlates with increased cell proliferation, invasion, metastasis, chemoresistance and poor prognosis (Chu et al., 2022; Liao et al., 2022). A growing body of research indicates that THBS2 functions as an emerging modulator of the TME, integrating stromal remodeling with anti-angiogenic signaling to dictate the trajectory of primary tumor expansion and metastatic dissemination (Liao et al., 2022). This “THBS2 paradox” suggests that while the protein is potent angiogenesis inhibitor, it has been implied to promote alternative vascularization and survival strategies that make the tumor more resilient to standard therapies (Zhang et al., 2025). The current review highlights the emerging pro-tumorigenic role of THBS2 protein.

## 2 Structural architecture and key molecular interactions

Understanding the functional versatility of THBS2 requires an analysis of its structural architecture. Each subunit of the THBS2 homotrimer consists of an N-terminal domain, an oligomerization domain, a procollagen homology (von Willebrand factor type C) domain, three thromobospondin type I repeats (TSR1–TSR3), three Type II (EGF-like) repeats, seven Type III calcium-binding repeats, and a C-terminal L-type lectin domain as depicted in Figure 1 (Yang et al., 2000; Carlson et al., 2005). These highly conserved domains enable THBS2 to interact with a wide range of ECM proteins, cell-surface receptors, and proteases, thereby regulating multiple physiological and pathological processes. The N-terminal domain mediates THBS2 to the ECM through interactions with heparin, heparan sulfate proteoglycans, and fibronectin, thereby regulating matrix assembly, cell adhesion, and migration. The adjacent α-helical coiled-coil oligomerization domain mediates interchain disulfide bond formation, assembling three THBS2 monomers into a stable homotrimer. The procollagen homology (von Willebrand factor type C) domain interacts with fibrillar collagens and other ECM proteins to regulate collagen fibrillogenesis, matrix stability, and tissue architecture (Yang et al., 2000; Carlson et al., 2005; Adams and Lawler, 2011; Huang et al., 2023).

**FIGURE 1:**
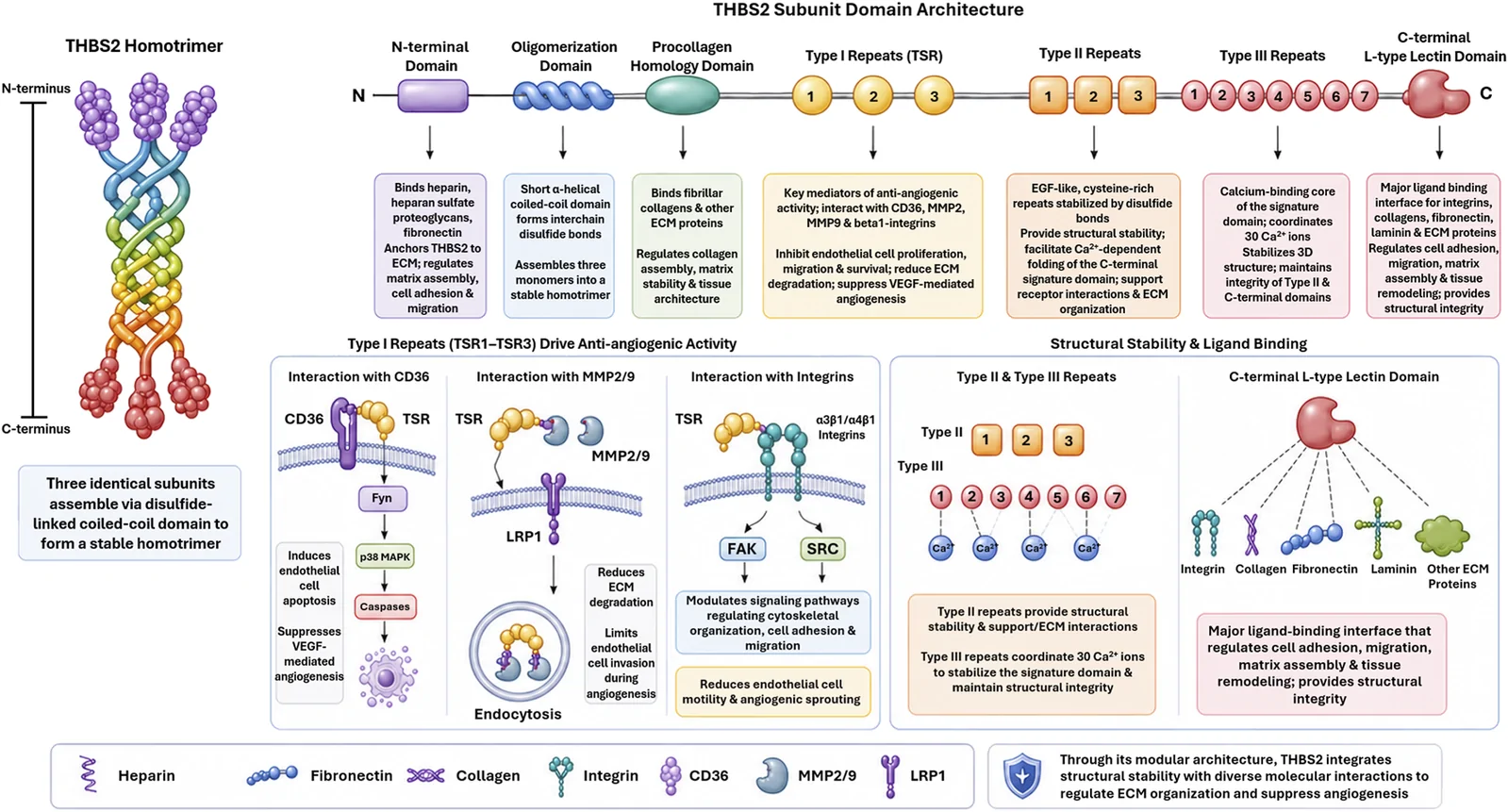
Structural organization and functional domains of Thrombospondin-2 (THBS2). THBS2 is a disulfide-linked homotrimer in which each subunit consists of an N-terminal domain, oligomerization domain, procollagen homology domain, three type I thrombospondin repeats (TSR1–TSR3), three type II EGF-like repeats, seven type III calcium-binding repeats, and a C-terminal L-type lectin domain. The N-terminal and procollagen domains mediate extracellular matrix (ECM) interactions and collagen organization, while the type I repeats may inhibit angiogenesis through interactions with CD36, MMP2/MMP9, and β1-containing integrins. The type II and type III repeats maintain structural stability through conserved disulfide bonds and Ca^2+^ binding, respectively, whereas the C-terminal L-type lectin domain binds multiple ECM ligands to regulate cell adhesion, migration, and matrix remodeling.

The three Type I repeats constitute the principal anti-angiogenic region of THBS2. These conserved cysteine-rich domains interact with several cell-surface receptors and extracellular proteins, including CD36, matrix metalloproteinases (MMP2 and MMP9), and β1-containing integrins, to regulate endothelial cell behavior and ECM remodeling (Yang et al., 2000; Adams and Lawler, 2011; Zhao et al., 2025). Through its conserved TSRs, THBS2 binds CD36 on endothelial cells and exerts anti-angiogenic effects that are believed to involve signaling pathways similar to those characterized for THBS1, including the Fyn kinase, p38 Mitogen-Activated protein kinase (MAPK), and caspase-dependent endothelial apoptosis (Dawson et al., 1999; Jiménez et al., 2000; Simantov et al., 2005; Huang et al., 2023). In addition, THBS2 antagonizes VEGF-mediated angiogenic signaling by reducing endothelial responsiveness to pro-angiogenic stimuli and limiting endothelial cell proliferation and migration. Moreover, the TSRs bind MMP2 and MMP9, promoting their low-density lipoprotein receptor-related protein 1 (LRP1) mediated endocytosis and clearance from the extracellular space, thereby reducing matrix degradation and limiting endothelial cell invasion during angiogenesis (Yang et al., 2000; Krady et al., 2008). THBS2 also interacts with β1-containing integrins, modulating focal adhesion kinase (FAK) and Src-dependent signaling to regulate cell adhesion, cytoskeletal organization, and migration (Adams and Lawler, 2011; Liu et al., 2020; Yao et al., 2026).

Following the Type I repeats, THBS2 contains three Type II repeats composed of epidermal growth factor (EGF)-like, cysteine-rich domains stabilized by multiple disulfide bonds. The Type II repeats contribute predominantly to the structural stability of THBS2 by supporting the calcium-dependent folding of the conserved C-terminal signature domain, facilitating receptor interactions, and ECM organization (Carlson et al., 2005). Immediately downstream, the seven Type III repeats form the calcium-binding core of the signature domain. This core coordinates approximately 30 Ca^2+^ ions, which stabilize the overall three-dimensional structure and maintain the integrity of the adjacent Type II repeats (Carlson et al., 2005; Huang et al., 2023). The C-terminal domain forms the terminal region of the conserved THBS2 signature domain. Functionally, this C-terminal domain serves as a major ligand-binding region. It binds with integrins, collagens, fibronectin, laminin, and other ECM proteins. Through these interactions, the domain regulates cell adhesion, migration, matrix assembly, and tissue remodeling (Carlson et al., 2005; Adams and Lawler, 2011). While proteins such as collagen and elastin provide a physical structural scaffold, THBS2 instead acts as a functional regulator of the communication between cells and the ECM (Bornstein, 2001; Murphy-Ullrich and Sage, 2014). In cancer, dysregulation of these cell–matrix interactions contribute to tumor progression in a context-dependent manner. Depending on the tumor type, cellular origin, receptor repertoire, and composition of the TME, THBS2 can either suppress angiogenesis and tumor growth or promote ECM remodeling, invasion, metastasis, and immune evasion (Murphy-Ullrich and Sage, 2014; Zhao et al., 2025).

## 3 Pro-tumorigenesis hallmarks involving Thrombospondin-2

### 3.1 THBS2 and angiogenesis paradox

Over several decades, THBS2 has been regarded as an endogenous angiogenesis inhibitor antagonizing vascular endothelial growth factor (VEGF) activity and regulating endothelial cell proliferation, migration, apoptosis and survival (Lawler and Lawler, 2012). However, recent studies have conceded its sole angiogenesis inhibitory function and indicate tumor specific proangiogenic role as depicted in Figure 2. Mechanistic studies by Zhang and colleagues demonstrated that THBS2 enhanced angiogenesis by upregulation of crucial angiogenesis genes, namely, vascular endothelial growth factor A (VEGFA), fibroblast growth factor 2 (FGF2), platelet endothelial cell adhesion molecule 1 (PECAM1), and fms-related tyrosine kinase 1 (FLT1) (Zhang et al., 2025). A systematic pan cancer analysis by Liu and colleagues established a robust positive association of THBS2 expression with tumor angiogenesis and concurrent suppression of DNA repair, cell cycle progression and DNA replication pathways (Liu et al., 2023). A compelling study on papillary thyroid cancer established that the chemokine CXCL16 is an upstream regulator of THBS2, which promotes THBS2 driven angiogenesis, ECM remodeling and invasion (Kim et al., 2019).

**FIGURE 2:**
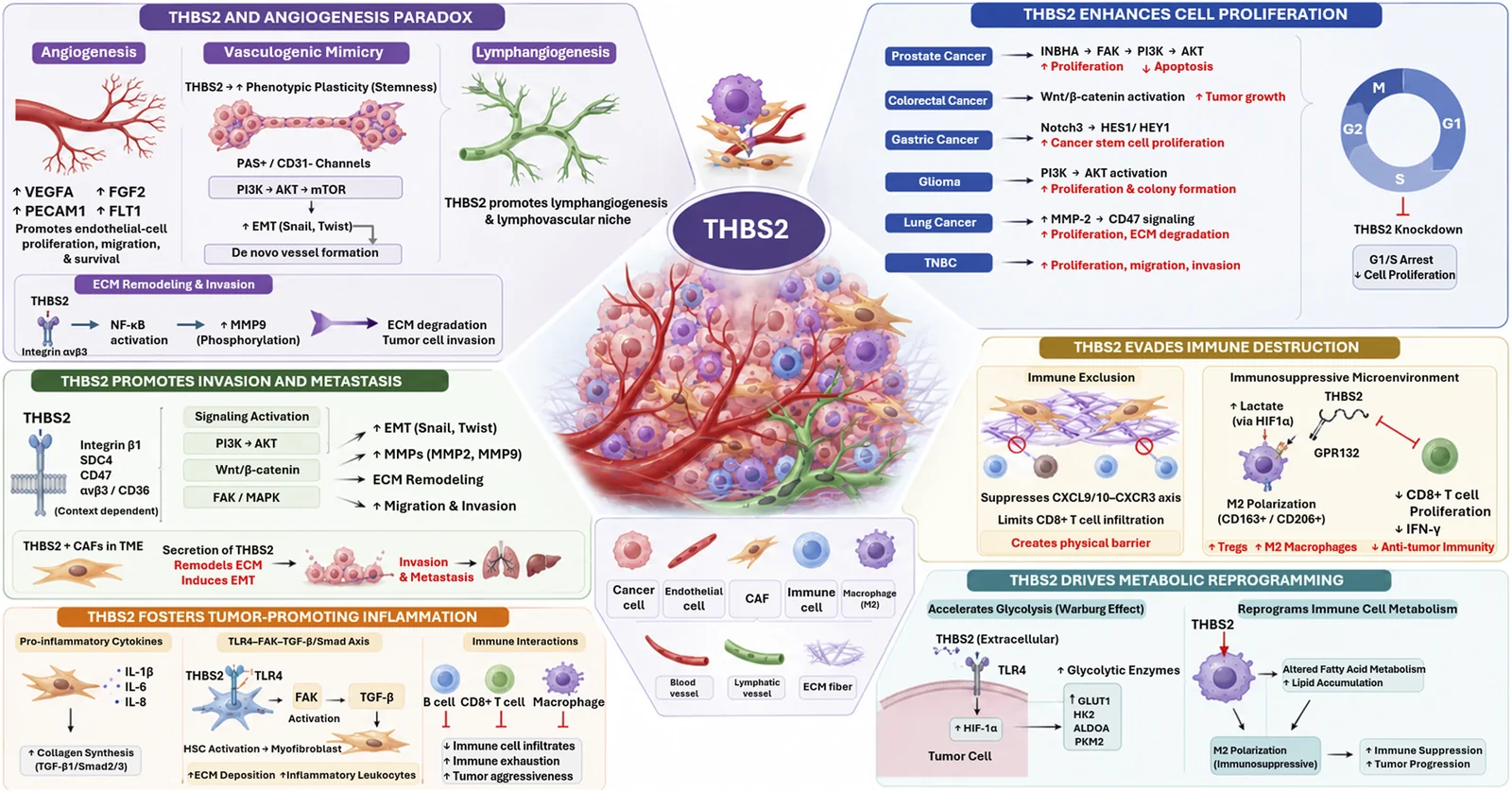
Multifaceted mechanisms of THBS2-mediated tumor progression. Overview of the six hallmarks influenced by Thrombospondin 2 (THBS2) overexpression in the TME. THBS2 actively coordinates angiogenesis (via VEGF), cell survival/proliferation (via CD47-PI3K/MAPK signaling), and metastatic dissemination (via EMT and ECM remodeling). Concurrently, it suppresses adaptive immunity (CD8^+^ T-cell evasion), fosters a pro-inflammatory niche, and reprograms metabolic pathways to favor enhanced glycolysis (Warburg effect) and lipid metabolism.

Another striking feature of tumor cells is their ability to survive by exhibiting vasculogenic mimicry (VM), which is the *de novo* formation of new fluid-conducting vessels in aggressive tumor cells. Bypassing classical angiogenesis, where new vessels arise from pre-existing ones, VM involves the trans-differentiation or lineage reprogramming of cancer cells owing to their stemness and plasticity. In clinical pathology, VM is characterized by a distinct CD31^-^/PAS^+^ phenotype identified by Periodic Acid–Schiff (PAS) staining. PAS positivity reflects the presence of tumor cell-derived, basement membrane-like ECM forming vessel-like channels, rather than endothelial cell-lined vasculature (Qiao et al., 2015; Huang et al., 2023). THBS2 intricately facilitates VM by positively regulating phenotypic plasticity, a stem cell like attribute also observed in cancer cells. Phenotypic plasticity, an emerging hallmark of cancer, is the reversible adoption of stem-like, mesenchymal, or drug-tolerant states in response to intrinsic or extrinsic microenvironmental cues, driving intratumoral heterogeneity, invasion, metastasis, and therapy resistance (Mehta and Stanger, 2024). Recently, Chang and colleagues reported that THBS2 promotes cancer stem cell like properties by enhancing the expression of Notch3 and sustaining the expression of stemness markers, namely, CD44, Nanog, OCT4, and SOX2) (Chang et al., 2024). To meet the demand of the growing cells, THBS2 drives VM by inducing the PI3K/AKT/mTOR signaling pathway which is central to cellular growth, survival, plasticity, and immune modulation (Huang et al., 2023). The activation leads to upregulation of epithelial-mesenchymal transition (EMT) associated transcription factors like snail and twist which eventually transform stationary polar epithelial cells into invasive mesenchymal cells (Jiang et al., 2025).

Diverging from previous roles, group A thrombospondins (THBS1 and THBS2) are also implied in promoting lymphangiogenesis, contributing to the tumor-associated lymphovascular niche in intrahepatic cholangiocarcinoma (Corbella et al., 2024). Albeit recombinant THBS2 functions as a more powerful inducer of cellular invasion and commits intrahepatic cholangiocarcinoma cells to an aggressive mesenchymal-like phenotype with higher intensity than recombinant THBS1. In addition to the morphological transformation of tumor cells to vascular phenotypes, THBS2 also regulates the phosphorylation of MMPs for ECM remodeling. Upon binding with integrin αvβ3, THBS2 triggers a cascade of reactions activating NF-κB to stimulate MMP9 production favoring tumor cell migration and tissue remodeling (Liu et al., 2020). Overall, THBS2 supports the vasculature of growing tumor cells collectively by promoting angiogenesis, vasculogenic mimicry and lymphangiogenesis, particularly in aggressive cancers.

### 3.2 THBS2 enhances cell proliferation

Recent studies have also highlighted the oncogenic role of THBS2 in malignant tissues correlating THBS2 upregulation to accelerated proliferative capacity. Latest study by Yao and colleagues, corroborates the oncogenic potential of THBS2 in prostate cancer. Their report provides evidence that upregulated THBS2 drives cell proliferation and inhibits apoptosis by activating the inhibin subunit beta A-dependent focal adhesion kinase (FAK)/phosphatidylinositol 3-kinase (PI3K)/protein kinase B (AKT) signaling pathway (Yao et al., 2026). However, in colorectal cancer, high THBS2 expression drives tumor growth by activating the canonical Wnt/β-catenin signaling pathway whereas its knockdown induces G1/S cell cycle arrest and inhibition of cell proliferation (Qu et al., 2022). Moreover, in gastric cancer, THBS2 regulates Notch3, HES1, and HEY1 and activates the Notch signaling pathway to augment cancer stem cell proliferation. Besides, lentiviral-mediated shRNA silencing of THBS2 impedes proliferation and reduces *in vivo* tumorigenesis in subcutaneous gastric cancer xenograft models (Chang et al., 2024). Similarly, siRNA mediated THBS2 silencing decreased invasive, migratory and proliferative abilities in triple-negative breast cancer (TNBC) cells *in vitro* (Lin et al., 2022). Consistent with these findings, Huang and colleagues concluded that by activation of the PI3K/AKT signaling pathway, THBS2 promotes cell growth, proliferation and colony formation in glioma while its knockdown leads to cell cycle arrest (Huang et al., 2022). Moreover, THBS2 knockdown also reduces A549 lung cancer cell proliferation and is associated with poor prognosis in aggressive lung adenocarcinoma patients (Weng et al., 2016). A pan-cancer analysis by Liao and colleagues established that THBS2 serves as a multi-functional bridge between the ECM and immune infiltrating cells(Liao et al., 2022). Mechanistically, THBS2 drives ECM remodeling by directly augmenting MMP-2 expression, which accelerates matrix degradation and tumor cell migration. Concurrently, it coordinates immune-related effects by regulating the CD47-mediated signaling pathway, ultimately promoting tumor proliferation and establishing a protective immune-suppressive microenvironment (Liao et al., 2022). To sum it up, overexpressed THBS2 steers uncontrolled cell proliferation acting as an oncogenic driver, particularly in epithelial cancers.

### 3.3 THBS2 promotes invasion and metastasis

In addition to pro angiogenesis and proliferative potential, THBS2 has also been identified as a promotor of cancer cell invasion and metastasis, providing favorable TME by ECM remodeling and inducing EMT. THBS2 knockout studies have established its pro-metastatic role in skin cutaneous melanoma wherein THBS2 modulates MMPs to degrade the basement membrane allowing tumor cells to detach and infiltrate the surrounding stroma. Functional assays have not only confirmed that THBS2 promotes invasion and hepatic migration of melanoma cells but also demonstrated its multifunctional role in ECM remodeling, positively correlated with ECM structural proteins (LAMA4, COL1A1, and COL4A1) and MMPs (MMP2 and MMP9) (Zhang et al., 2025). Besides, THBS2 interacts with integrin β1 to activate the PI3K/AKT signaling pathway to facilitate cell migration, invasion, and EMT in breast cancer (Qi et al., 2023). The evidence in other cancers also reasserts the MMP regulatory function and subsequent metastasis as reported in colorectal, lung, gastrointestinal, prostate and osteosarcoma studies well-characterized via Wnt/β-catenin signaling, ECM remodeling, αvβ3/CD36-MAPK and FAK/PI3K/AKT activation (Liu et al., 2020; Chu et al., 2022; Li and Wu, 2023; Zhang et al., 2025; Li and Xie, 2026). Furthermore, THBS2-producing matrix Cancer-associated fibroblasts (CAFs) trigger phenotypic changes in TME enhancing EMT serving as critical drivers of progression in lung adenocarcinoma, colorectal, pancreatic, and gastric cancer (Nan et al., 2022; Li et al., 2025; Liu et al., 2025; Ren et al., 2025). To activate pro-tumorigenic signaling cascades CAF-derived THBS2 binds distinct tumor cell receptors such as syndecan 4 (SDC4) in lung cancer, CD47 in colorectal cancer, integrin αvβ3/CD36 in pancreatic cancer and CD36 in gastric cancer. Functional studies consistently show THBS2 knockdown suppresses invasion and migration while overexpression enhances these phenotypes. Taken together, THBS2 promotes invasion and metastasis through multiple interconnected mechanisms including enhanced angiogenesis and proliferation, ECM remodeling, and activation of pro-metastatic signaling pathways across diverse cancer types.

### 3.4 THBS2 evades immune destruction

Several recent studies have reported that THBS2 suppresses anti-tumor immunity through multiple mechanisms, enabling tumors to evade immune destruction. A recent study indicates that THBS2-expressing mCAFs regulate the immune-exclusionary environment in fibrotic colorectal cancer and evade immune destruction by limiting CD8^+^ T cell infiltration establishing a physical barrier to immune infiltration. Experimental studies using orthotopic model with desmoplastic tumor organoids showed that deletion of THBS2 increased intratumoral CD8^+^ T cells (Iwane et al., 2025). Their findings clearly demonstrate the ability of THBS2 in maintaining an exclusionary barrier for CD8^+^ T cells by suppressing the CXCL9/10-CXCR3 signaling axis, crucial for T cell recruitment, through its inhibitory effects on dendritic cells and macrophages. Another study in CT26 colorectal cancer cells by Liu and colleagues demonstrates that THBS2 overexpression promotes HIF1α nuclear translocation and enhanced lactate production through anaerobic glycolysis, induces M2 (anti-inflammatory) polarization of macrophages, characterized by increased CD163 and CD206 expression, mediated through lactate-GPR132 signaling (Liu et al., 2023). These M2-polarized macrophages significantly suppressed CD8^+^ T cell proliferation and reduced IFN-γ release. Furthermore, *in vivo* assays in CT26-THBS2 tumor-bearing mice showed significantly increased tumor volume with higher infiltration of regulatory T cells and M2-polarized macrophages compared to controls. CD8^+^ T cell proliferation was considerably reduced in THBS2-overexpressing tumors representing a well-characterized immune evasion pathway where THBS2 creates an immunosuppressive microenvironment to evade immune destruction. A comprehensive pan-cancer analysis by Liao and colleagues followed by experimental validation reasserts THBS2’s role as a bridge between ECM and immune infiltration (Liao et al., 2022). THBS2 showed positive correlations with StromalScore in 30 cancer types and ImmuneScore in 19 cancer types, indicating its dual role in ECM remodeling and immune regulation. Additionally, THBS2 expression was also positively correlated with tumor-promoting immune cells such as M2 macrophages, neutrophils, monocytes and negatively correlated with tumor-suppressing cells activated NK cells, T follicular helper cells indicating immunosuppressive function. Therefore, THBS2 effectively creates a physical and chemical barrier to exclude or suppress immune cells from destroying the cancerous cells.

### 3.5 THBS2 fosters tumor-promoting inflammation

Beyond creating an immune exclusionary physical and chemical barrier, THBS2 fuels chronic, pro-tumorigenic inflammation by binding to distinct cellular receptors and activating downstream pro-survival signaling cascades. A recent study by Yu and colleagues provides robust evidence of THBS2’s pro inflammatory effects wherein THBS2 overexpression not only significantly promotes inflammatory secretome including IL-1β, IL-6, and IL-8 in fibroblasts but also enhances collagen synthesis through the TGF-β1/Smad2/3 signaling pathway (Yu et al., 2025). Another interesting report by Zhang and colleagues (2024) demonstrates that THBS2 drives hepatic fibrosis and tumor-promoting inflammation by binding to toll-like receptor 4 (TLR4) on hepatic stellate cells, triggering FAK activation and the TGF-β/Smad signaling pathway. This axis fosters a pathogenic positive-feedback loop, promoting HSC-to-myofibroblast differentiation, excessive ECM deposition, and the recruitment of inflammatory leukocytes to establish a pro-tumorigenic microenvironment (Zhang et al., 2024). Besides, in early-stage lung adenocarcinoma studies, THBS2+ CAFs interact with B cells, CD8^+^ T lymphocytes, and macrophages within the TME and promote tumor aggressiveness by suppressing antitumor immunity, decreasing immune cell infiltrates, and increasing immune exhaustion markers (Yang et al., 2022).

### 3.6 THBS2 drives metabolic reprogramming

Emerging evidence indicates that THBS2 contributes to metabolic reprogramming in the TME by promoting tumor progression and immune evasion. In CRC, extracellular THBS2 has been reported to interact with TLR4 on the surface of tumor cells. This interaction is reported to upregulate HIF-1α protein levels and increase expression of key glycolytic enzymes (GLUT1, HK2, ALDOA, PKM2, LDHA) enabling metabolic reprogramming in tumor cells. The upregulation of the rate-limiting glycolytic enzymes shift cellular metabolism away from oxidative phosphorylation towards aerobic glycolysis. Commonly referred to as the Warburg effect, this metabolic reprogramming accelerates energy production to sustain rapid cell proliferation and tumor growth (Xu et al., 2020). In addition to its effects on tumor cell metabolism, THBS2 has been implicated in the metabolic regulation of immune cells. In lung adenocarcinoma, THBS2 primarily reprograms fatty acid metabolism pathways in macrophages driving their transformation into the immunosuppressive and tumor-promoting M2 subtype (Weng and Zhu, 2025). Mechanistically, THBS2 disrupts fatty acid metabolism pathways within infiltrating immune cells to accelerates intracellular lipid accumulation and alters downstream signaling cascades. Consequently, this metabolic shift drives the polarization of uncommitted macrophages toward a pro-tumorigenic, M2-like phenotype. Thus, via metabolic reprogramming THBS2 secures critical energy substrates for accelerated tumor progression while constructing a protective niche that shields cancer cells from immune-mediated destruction.

## 4 THBS2 and resistance to therapy

A growing body of literature suggests that THBS2 plays a significant role in promoting resistance to therapy particularly in CRC, TNBC and lung adenocarcinoma through distinct mechanisms. Recently, Iwane and colleagues demonstrated that targeting THBS2 transforms immunotherapy-resistant fibrotic colorectal cancers into treatment-responsive tumors as ascertained by the dramatic response to immune checkpoint inhibitors in THBS2-deficient tumors. THBS2 loss creates a favorable immune environment by disrupting the exclusionary barrier, increasing CD8^+^ T cell infiltration with increased exhaustion markers (PD-1, CTLA-4), making tumors highly susceptible to checkpoint blockade. Anti-PD-1 antibody treatment achieved complete pathological remission in all treated THBS2-knockout mice indicating that THBS2 plays a pivotal role in resistance to therapy (Iwane et al., 2025). Another recently published regulatory mechanism in colon cancer involves MARCHF1 mediated SULF1 degradation which disrupts the SULF1-THBS2 interaction (Li and Xie, 2026). This disruption blocks THBS2/TGF-β/SMAD2/3 signaling and leads to subsequent reversal of metastasis and pronounced 5-FU resistance.

Besides, THBS2-positive CAFs secrete COL8A1, which interacts with the ITGB1 receptor on resistant cancer cells, activating PI3K-AKT signaling and stimulating EMT eventually promoting oxaliplatin resistance in colorectal cancer (Zhou et al., 2024). THBS2 serves as a biomarker for chemotherapy resistance in TNBC, where elevated serum THBS2 levels at surgery correlate with poorer response to neoadjuvant chemotherapy with lower pathological complete response rates (OR = 0.88, p = 0.020), and histological THBS2 independently predicts poor disease-free survival (HR = 2.21, p = 0.007) and overall survival (HR = 2.07, p = 0.026) (Lin et al., 2022). In early-stage lung adenocarcinoma, high THBS2 expression correlates with poor response to immunotherapies and shortened post-treatment survival in patients. THBS2 levels in peripheral plasma were associated with short recurrence-free survival, suggesting potential as a liquid biopsy biomarker (Yang et al., 2022). These reports underscore THBS2 as a major obstacle to successful cancer therapy and an excellent marker for both diagnostic and prognostic purposes.

## 5 Determinants of the context-dependent functions of THBS2

The role of THBS2 in cancer is increasingly recognized as being highly dependent on the biological context in which it is expressed. One of the major factors influencing its function is the source of THBS2 within the tumor tissue. In healthy tissues, THBS2 is secreted by fibroblasts to regulate collagen fibrillogenesis, maintain ECM organization, and inhibit angiogenesis (Yang et al., 2000). On the contrary, during malignant transformation, activated CAFs become a dominant source of THBS2. CAF-derived THBS2 is associated with collagen remodeling, matrix stiffening, and desmoplastic stromal remodeling. These processes are linked to enhanced tumor invasion and metastatic dissemination as reported for CRC and lung adenocarcinomas. Emerging evidence suggests that THBS2 may participate in stromal signaling networks involving TGF-β and other profibrotic mediators to promote fibrosis, ECM remodeling, and immune suppression (Zhang et al., 2024; Zhou et al., 2024; Iwane et al., 2025; Ren et al., 2025; Zhao et al., 2025).

The repertoire of receptors present on the neighboring cells also dictate THBS2’s cellular response. Engagement of CD36 by the Type I repeats in endothelial cells activates the Fyn–p38 MAPK–caspase signaling cascade which results in endothelial apoptosis and inhibition of neovascularization (Dawson et al., 1999; Jiménez et al., 2000). In contrast, tumor cells expressing integrins such as αvβ3, α3β1, and other β1-containing integrins respond to THBS2 through activation of FAK- and Src-associated signaling pathways. Consequently, these pathways promote cell adhesion, migration, and survival (Bornstein, 2001; Carlson et al., 2005; Adams and Lawler, 2011). The balance between CD36-mediated anti-angiogenic signaling and integrin-mediated pro-survival signaling represents an important determinant of the biological outcome of THBS2 activity. The function of THBS2 also changes as tumors evolve. During the early stages of malignant transformation, THBS2 primarily restricts vascular growth and preserves tissue architecture. However, as the disease progresses, persistent hypoxia, chronic inflammation, and extensive remodeling of the ECM reshape the TME, altering receptor availability and signaling networks. THBS2 expression has been associated with EMT, immune evasion, invasion, and metastatic dissemination in several aggressive cancers (Murphy-Ullrich and Sage, 2014; Zhao et al., 2025). Taken together, these observations imply that the transition of THBS2 from a tumor-suppressive to a tumor-promoting factor does not result from intrinsic alterations in the protein itself, but from dynamic changes in the cellular and extracellular microenvironment that dictate receptor engagement, signaling outcomes, and biological function.

## 6 THBS2-targeted therapeutic strategies

THBS2 has emerged as a potential therapeutic target since it participates in multiple processes that influence tumor development and progression. Although no THBS2-specific therapeutic agent has reached clinical application yet, several strategies have demonstrated promising preclinical efficacy. In head and neck squamous cell carcinoma (HNSCC), the mitochondrial metabolism inhibitor CPI-613 (devimistat) selectively disrupts mitochondrial energy metabolism, inducing endoplasmic reticulum stress and activation of the X-box binding protein 1 (XBP1) signaling pathway. Activated XBP1 was shown to repress THBS2 transcription, resulting in reduced THBS2 expression within the TME. This was reported to promote the infiltration and activation of CD8^+^ cytotoxic T cells, thereby alleviating tumor-associated immunosuppression and enhancing antitumor immunity (Yang et al., 2025). These findings identify the CPI-613–XBP1–THBS2–CD8^+^ T-cell axis as a promising therapeutic strategy for remodeling the immune microenvironment in HNSCC (Zhao et al., 2025). In addition, peptide-based approaches targeting thrombospondin signaling have attracted considerable interest. Anti-angiogenic peptides such as ABT-510 and ABT-898, which were developed from the TSR domain of THBS1, inhibit angiogenesis through CD36-dependent signaling (Garside et al., 2010). Although these agents are not direct THBS2-targeted therapeutics, the structural and functional conservation between the TSR domains of THBS1 and THBS2 raises the possibility that THBS2-derived anti-angiogenic mimetics could be developed in the future. (Dawson et al., 1999; Garside et al., 2010).

Recent study by Zhao and colleagues indicates that THBS2 modulation may improve the efficacy of immune checkpoint blockade (Zhao et al., 2025). In CRC, THBS2-deficient mice treated with anti-PD-1 antibodies exhibited significantly enhanced antitumor responses. THBS2 deficiency increased the infiltration and activation of CD8^+^ cytotoxic T cells. Moreover, several animals achieved complete pathological remission. Thus, in CRC, THBS2 contributes to the establishment of an immunosuppressive TME limiting the effectiveness of checkpoint inhibitors. These findings provide a strong rationale for combining THBS2-targeted strategies with immune checkpoint inhibitors. Particularly in tumors with elevated THBS2 expressions, this strategy can improve therapeutic outcomes. Nevertheless, these approaches remain at the preclinical stage, and further studies are warranted to develop selective THBS2-targeted therapeutics and establish their safety and efficacy in clinical settings (Zhao et al., 2025). The context-dependent functions of THBS2 across different tumor types necessitate careful evaluation of patient selection and therapeutic strategies, as THBS2 can exert both tumor-suppressive and tumor-promoting effects depending on the cellular and microenvironmental context.

## 7 Conclusion

Almost 50% of cancer deaths worldwide could potentially be averted through better primary prevention, enhanced early detection, and more effective curative treatment strategies (Langselius et al., 2026). The path toward mitigating the global cancer burden requires a paradigm shift from reactive “late-stage” management to early detection and development of curative strategies with novel targets to cope with resistance to therapy. THBS2 is an active architect of the tumor’s survival strategy which governs angiogenesis and the structural formation of VM along with its emerging pivotal role in several biochemical pathways driving therapeutic resistance. Besides, THBS2 overexpression reflects a group of tumors with more aggressive behaviour and poor outcome. THBS2 provides aggressive tumors with the tools needed to survive in a hostile environment and resist medical intervention. Therefore, elevated THBS2 levels can be used as a biomarker for not only for diagnosis but also for patient stratification and selection of treatment regimen. Moreover, targeting it can overcome resistance to traditional chemo-therapeutic agents. Structure-based drug designing can be used to design small molecule inhibitors or peptides that block specific protein-protein interactions of THBS2, such as its interaction with MMPs or other stromal components (Damani Shah et al., 2019; Shah et al., 2020). Future research focusing on the clinical utility of this interesting target is warranted especially in other high incidence and high mortality cancers such as lung, stomach, liver, and oral cancer.

## Glossary

ABT-510: Anti-angiogenic Thrombospondin-1 Mimetic Peptide 510
ABT-898: Anti-angiogenic Thrombospondin-1 Mimetic Peptide 898
AKT: Protein Kinase B
ALDOA: Aldolase A
CA19-9: Carbohydrate Antigen 19-9
CAF: Cancer-Associated Fibroblast
Ca^2+^: Calcium Ion
CD31 (PECAM1): Platelet Endothelial Cell Adhesion Molecule 1
CD36: Cluster of Differentiation 36
CD44: Cluster of Differentiation 44
CD47: Cluster of Differentiation 47
CD163: Cluster of Differentiation 163
CD206: Cluster of Differentiation 206
CEBPB: CCAAT/Enhancer-Binding Protein Beta
COL1A1: Collagen Type I Alpha 1 Chain
COL4A1: Collagen Type IV Alpha 1 Chain
COL8A1: Collagen Type VIII Alpha 1 Chain
CRC: Colorectal Cancer
CT26: Colon Carcinoma Cell Line 26
CTLA-4: Cytotoxic T-Lymphocyte-Associated Protein 4
CXCL9: C-X-C Motif Chemokine Ligand 9
CXCL10: C-X-C Motif Chemokine Ligand 10
CXCL16: C-X-C Motif Chemokine Ligand 16
DNA: Deoxyribonucleic Acid
ECM: Extracellular Matrix
EGF: Epidermal Growth Factor
EMT: Epithelial–Mesenchymal Transition
ER: Endoplasmic Reticulum
FAK: Focal Adhesion Kinase
FGF2: Fibroblast Growth Factor 2
FLT1: Fms-Related Tyrosine Kinase 1 (VEGFR1)
GLOBOCAN: Global Cancer Observatory
GLUT1: Glucose Transporter 1
GPR132: G Protein-Coupled Receptor 132
HES1: Hes Family BHLH Transcription Factor 1
HEY1: Hes Related Family BHLH Transcription Factor with YRPW Motif 1
HIF-1α: Hypoxia-Inducible Factor 1 Alpha
HK2: Hexokinase 2
HNSCC: Head and Neck Squamous Cell Carcinoma
HR: Hazard Ratio
IFN-γ: Interferon Gamma
IL-1β: Interleukin 1 Beta
IL-6: Interleukin 6
IL-8: Interleukin 8
INHBA: Inhibin Subunit Beta A
ITGB1: Integrin Beta 1
LAMA4: Laminin Subunit Alpha 4
LDHA: Lactate Dehydrogenase A
LOX: Lysyl Oxidase
LRP1: Low-Density Lipoprotein Receptor-Related Protein 1
MAPK: Mitogen-Activated Protein Kinase
MARCHF1: Membrane Associated Ring-CH-Type Finger 1
mCAFs: Myofibroblastic Cancer-Associated Fibroblasts
MMP2: Matrix Metalloproteinase 2
MMP9: Matrix Metalloproteinase 9
mTOR: Mammalian Target of Rapamycin
NF-κB: Nuclear Factor Kappa B
Notch3: Neurogenic Locus Notch Homolog Protein 3
OCT4: Octamer-Binding Transcription Factor 4
OR: Odds Ratio
PAS: Periodic Acid–Schiff
PD-1: Programmed Cell Death Protein 1
PDAC: Pancreatic Ductal Adenocarcinoma
PI3K: Phosphatidylinositol 3-Kinase
PKC: Protein Kinase C
PKM2: Pyruvate Kinase M2
PLC: Phospholipase C
SDC4: Syndecan 4
SMAD: Small Mothers Against Decapentaplegic
SOX2: SRY-Box Transcription Factor 2
SULF1: Sulfatase 1
TCA Cycle: Tricarboxylic Acid Cycle
TGF-β: Transforming Growth Factor Beta
THBS1: Thrombospondin-1
THBS2: Thrombospondin-2
TLR4: Toll-Like Receptor 4
TME: Tumor Microenvironment
TNBC: Triple-Negative Breast Cancer
TSR: Thrombospondin Type I Repeat
TSR1–TSR3: Type I Thrombospondin Repeats 1–3
TSPs: Thrombospondins
VEGF: Vascular Endothelial Growth Factor
VEGFA: Vascular Endothelial Growth Factor A
VEGFR1: Vascular Endothelial Growth Factor Receptor 1
VM: Vasculogenic Mimicry
XBP1: X-Box Binding Protein 1
